# Depressive Symptoms, Perceived Stress, Self-Compassion and Nonsuicidal Self-Injury Among Emerging Adults: An Examination of the Between and Within-Person Associations Over Time

**DOI:** 10.1177/21676968211029768

**Published:** 2021-07-09

**Authors:** Holly Boyne, Chloe A. Hamza

**Affiliations:** 1 Applied Psychology and Human Development, Ontario Institute for Studies in Education, University of Toronto, Ontario, Canada

**Keywords:** nonsuicidal self-injury, emerging adulthood, depression, stress, self-compassion

## Abstract

Many emerging adults report experiencing mental health challenges (e.g., depressive symptoms and perceived stress) during the transition to university. These mental health challenges often coincide with increased engagement in nonsuicidal self-injury (NSSI; e.g., self-cutting or burning without lethal intent), but longitudinal research exploring the nature of the associations among depressive symptoms, perceived stress, and NSSI are lacking. In the present study, it was examined whether depressive symptoms and perceived stress predicted increased risk for NSSI over time (or the reverse), and whether these effects were mediated or moderated by self-compassion. The sample consisted of 1,125 university students (*M*age = 17.96 years, 74% female), who completed an online survey three times in first year university. A random intercept cross-lagged panel model revealed that higher depressive symptoms, perceived stress, NSSI, and lower self-compassion often co-occurred, but only NSSI predicted increased perceived stress over time. Theoretical and practical implications are discussed.

The post-secondary years are a significant period of change and transition for emerging adults (ages 18–25), and navigating new roles and responsibilities may be challenging for some students (e.g., living away from home for the first time, managing new academic pressures, etc.) (Arnett, 2014; Arnett et al., 2014). Although some emerging adults cope well during the transition to post-secondary school, other emerging adults have more difficulty. In a recent nationally representative study of post-secondary students in Canada, it was found that 88% of post-secondary students reported feeling overwhelmed by all they had to do, 76% reported that they felt very sad, and 52% reported feeling so depressed it was difficult to function (American College Health Association, 2019). Risk for suicidal thoughts and behaviors also increases during this time (Mortier et al., 2017), and suicide is the second leading cause of death among young persons ages 15–24 in Canada (Statistics Canada, 2020). Given that the early university years represent an important period of development in which mental health trajectories are set or altered in enduring ways (e.g., 75% of mental health disorders first have their onset before age 24 years) (Kessler et al., 2005; Statistics Canada, 2020), supporting emerging adults during the post-secondary years is critical to promote well-being across the lifespan.

One behavior that is reflective of mental health challenges among post-secondary students is nonsuicidal self-injury (NSSI), which is defined as the direct, deliberate destruction or alteration of body tissue in the absence of suicidal intent (e.g., self-cutting or burning) (International Society for the Study of Self-Injury, 2018). NSSI does not include behaviors that are socially sanctioned (e.g., tattooing or piercing), or that are a result of severe developmental delays or disabilities (American Psychiatric Association, 2013). As many as 20% of post-secondary students report engaging in NSSI (Kiekens et al., 2018a; Muehlenkamp & Brausch, 2019; Swannell et al., 2014), and these rates appear to be increasing (Wester et al., 2018; Xiao et al., 2017). Further, the rates of NSSI among emerging adults in post-secondary school are two times higher than same-aged peers who do not attend post-secondary school, suggesting that students may represent a unique at risk group (Swannell et al., 2014).

NSSI often coincides with elevated intra- and interpersonal distress, such that individuals who engage in NSSI report higher levels of emotion dysregulation (i.e., difficulty modulating emotional responses) (You et al., 2018), and negative affect (Victor & Klonsky, 2014), as well as higher levels of exposure to interpersonal stressors (e.g., interpersonal conflict, rejection, and criticism) (Turner et al., 2016; Victor et al., 2018). These findings have led researchers to conclude that NSSI primarily serves as a form of emotion coping behavior, used to mitigate mounting intra- and interpersonal distress (Taylor et al., 2018). Although NSSI and suicidal behavior can be differentiated on the basis of non-lethal intent (Hamza et al., 2012), there is evidence that NSSI engagement is associated with increased rates of suicidal ideation and attempts among post-secondary students (Kiekens et al., 2018b). As a result, understanding the processes through which NSSI develops is necessary to inform prevention and intervention efforts on campuses, and may circumvent risk for later suicidal behaviors.

## Depressive Symptoms, Stress, and NSSI

Research has consistently shown that mental health challenges increase during the post-secondary years, particularly depressive symptoms and stress (Salmela-aro et al., 2008; Tanner & Arnett, 2011). Although these may co-occur with NSSI, it is also possible that depressive symptoms and stress may contribute to the increased rates of NSSI among students (Wester et al., 2018). For example, there are several studies that have demonstrated that depressive symptoms and NSSI are positively associated cross-sectionally (Glenn & Klonsky, 2011; Hankin & Abela, 2011; Kokaliari et al., 2017), and there is also some indication that depressive symptoms may predict NSSI onset and engagement over time among adolescents and young adults (Garisch & Wilson, 2015; Hankin & Abela, 2011). In a longitudinal study following a large cohort of students through their undergraduate degree, depression diagnosis before and during post-secondary school were independently associated with lifetime NSSI engagement (Wilcox et al., 2012). Further, a depression diagnosis before 3rd year of post-secondary school predicted NSSI engagement and NSSI frequency in 4th year (Wilcox et al., 2012). Sadness also has been shown to predict later NSSI urges in studies using daily diary assessments (Bresin et al., 2013).

Exposure to stressful life events and subjective experiences of stress (i.e., perceived stress) also have been connected to NSSI (Kokaliari et al., 2017; Miller et al., 2019). In a recent meta-analysis, people who reported more significant life stress were found to have 33% higher odds of NSSI engagement (Liu et al., 2016). In studies specific to post-secondary students and young adults, greater numbers of stressful life events were related to increased NSSI engagement and frequency of NSSI (Ewing et al., 2019; Juanchan et al., 2019; Kiekens et al., 2019; Manca et al., 2014). Studies involving real-time assessment of stress and NSSI yield similar findings. In one study of young adults, perceived stress earlier in the day was associated with an increased likelihood of persistent NSSI thoughts and intense NSSI urges later in the day (Turner et al., 2019), and higher rates of perceived stress reduced the likelihood of successfully resisting NSSI urges within the same day (Turner et al., 2019). In other studies involving real-time sampling, perceived stress was associated with same-day NSSI engagement within the individual (i.e., higher than their average stress level predicted NSSI) (Kleiman et al., 2018; Miller et al., 2019), and between other participants (i.e., higher stress compared to their peers predicted NSSI) (Kleiman et al., 2018). These findings are consistent with stress-sensitivity theory that suggests that greater exposure to stressful life events may increase risk for NSSI (March-Llanes et al., 2017; Nock, 2010).

## The Role of Self-Compassion

Little attention has been given to why depressive symptoms and stress may be associated with heightened risk for NSSI. One possibility is that depressive symptoms and stress may negatively impact an individual’s self-beliefs, which in turn may lead to NSSI. In the Benefits and Barriers Model of NSSI, Hooley and Franklin (2017) posit that if an individual maintains a positive self-view, they may be less likely to engage in NSSI. If the positive self-view “barrier” is removed, an environment may be created in which the individual desires to escape unpleasant feelings, believes they are deserving of punishment, and develops greater identification with NSSI. Self-compassion involves taking a non-judgmental view of one’s thoughts and feelings, in which the individual can see their pain as part of a larger human experience, rather than a personal flaw (Neff, 2003). Experiencing depressive symptoms and stress may diminish self-compassion, leading one to regard themselves as damaged, needy, or interpersonally incompetent when experiencing negative mood states (Quirk et al., 2015; Xavier et al., 2016). Depressive symptoms and stress have been shown to be negatively associated with self-compassion in the literature (Bluth et al., 2016; Gilbert et al., 2011; Kaniuka et al., 2019; Kelliher-Rabon et al., 2018) and self-compassion has been shown to be negatively associated with NSSI engagement (Gregory et al., 2017). Further, cross-sectional work suggests that self-compassion may mediate the relations between negative affect and NSSI, parental closeness and NSSI, and childhood maltreatment and NSSI (Hasking et al., 2018; Jiang et al., 2017; Tarber et al., 2016).

Another possibility is that self-compassion may serve as a protective factor against NSSI, such that when individuals are high in self-compassion, they are less likely to engage in NSSI in response to mounting depressive symptoms and perceived stress (i.e., self-compassion could be a moderator of the associations between depressive symptoms, perceived stress, and NSSI). Theories on NSSI engagement have long underscored NSSI as a form of emotion coping (Klonsky & Glenn, 2009; Nock & Prinstein, 2004), and distress stemming from depressive symptoms and stress may lead to NSSI (Miller et al., 2019; Nock, 2010). As Hooley and Franklin (2017) highlight, having a positive self-view may create an environment where it is difficult to engage in NSSI, as the individual regards themself favorably and seeks to protect their body rather than deliberately inflict harm. Therefore, when distressed, students with high levels of self-compassion may select other methods of coping than NSSI, as the positive self-association represents a barrier to NSSI. Some studies have explored self-compassion as a potential moderating factor; in two cross-sectional studies, the association between depressive symptoms and NSSI was stronger among students with low self-compassion than among students with high self-compassion (Kaniuka et al., 2019; Xavier et al., 2016). However, given the lack of multi-wave longitudinal research on depressive symptoms, stress, self-compassion, and NSSI to date, it is difficult to understand if self-compassion is better conceptualized as a mediator or moderator of the processes through which depressive symptoms and stress may be associated with NSSI engagement.

## Study Purpose

There is a need to explore the longitudinal associations among depressive symptoms, perceived stress, self-compassion, and NSSI using multiple assessment waves to capture potential pathways to NSSI. Examining these associations using a multi-wave design affords the opportunity to disentangle between-person and within-person effects, which is necessary to capture temporal predictors of NSSI (Hamaker et al., 2015). For example, research suggests that there may be stable trait-like individual differences in depressive symptoms, perceived stress, self-compassion, and NSSI, such that individuals tend to be consistently higher or lower in these constructs, relative to others (Barrocas et al., 2014; Masselink et al., 2018; Miller et al., 2020; Neff, 2003). It is possible that these trait-like individual differences are associated across time (e.g., that individuals who are higher on depressive symptoms also tend to be higher on NSSI), rather than within-person temporal relations (e.g., higher depressive symptoms predict higher levels of NSSI engagement over time). By using an emerging analytic approach called random intercept cross-lagged panel modeling (RI-CLPM) (Hamaker et al., 2015), the present study focused on capturing within-person changes in perceived stress, depressive symptoms, and NSSI over time, taking into account between-person differences. To our knowledge, our study represents the first to examine the predictive effects of depressive symptoms and perceived stress on NSSI taking into account both between-person and within-person sources of variance. Additionally, we extended upon cross-sectional findings, to test whether self-compassion served as both a mediator and moderator of the within-person associations among depressive symptoms, perceived stress, and NSSI over time.

Another significant strength of the present study is that we also examined the direction of within-person effects among depressive symptoms, perceived stress, self-compassion, and NSSI. Although research to date has largely focused on the predictive effects of depressive symptoms and stress on NSSI, it is also possible that NSSI may lead to heightened depressive symptoms and stress over time. For example, theories of stress generation suggest that engaging in NSSI could lead to experiencing heightened stressors (e.g., interpersonal disruption) (Burke et al., 2015; March-Llanes et al., 2017). Further, engaging in NSSI may decrease an individual’s self-compassion (rather than the reverse), given that NSSI is a highly stigmatized behavior (Lloyd et al., 2018; Piccirillo et al., 2020). Experiences of guilt and shame following NSSI are commonly retrospectively reported among adolescents and young adults (Klonsky & Glenn, 2009; Laye-Gindhu & Schonert-Reichl, 2005), suggesting that NSSI may not only be impacted by self-compassion, but NSSI may impact one’s self-beliefs as well (Xavier et al., 2016). Using RI-CLPM, the present study addresses this gap in the literature by examining the nature of associations among depressive symptoms, perceived stress, self-compassion, and NSSI across time.

Based on existing research and theoretical models on NSSI, it was hypothesized that: 1) There would be stable, between-person differences in depressive symptoms, perceived stress, self-compassion, and NSSI (e.g., participants who scored higher on depressive symptoms relative to other participants at one time point, would score higher on depressive symptoms relative to other participants at other time points), and that these trait differences would be related (e.g., individuals with higher trait depressive symptoms, higher trait stress, and lower levels of self-compassion, on average, would score higher on trait NSSI); 2) It also was expected that at times when individuals reported higher levels of depressive symptoms, higher levels of perceived stress, and lower self-compassion (than their typical levels), they would also report higher than their typical levels of NSSI engagement; 3) There would be significant within-person associations between depressive symptoms, perceived stress, and NSSI (such that depressive symptoms and perceived stress would predict higher levels of NSSI over time, taking into account between-person differences). We also examined whether associations were bidirectional (which was more exploratory); and 4) Self-compassion would both mediate and moderate the within-person effects of depressive symptoms and perceived stress on NSSI over time, controlling for trait-like between-person differences in these constructs.

## Method

### Participants

Participants in this study included 1,125 1st year undergraduate students at a large Canadian university (74% female, 25% male, 1% other; M*age* = 17.96, *SD* = .69) who were part of a larger ongoing longitudinal research project examining stress and coping among emerging adults during the transition to university. The sample size was chosen to ensure sufficient statistical power for the proposed data analytic plan (a minimum of 200 for path analysis) (Kline, 2005), and to ensure a large number of students who engaged in NSSI were represented (approximately 200 students, or 20%; Swannell et al., 2014). In terms of ethnicity, the sample was 32% East Asian, 23% South Asian, 21% Caucasian, 6% Arab or West Asian, and 18% other including Black, West Indian, Latin American, and Filipino. Socioeconomic status was inferred by the mean level of education of participants’ parents, which fell between “some university” and a “university degree” for both mothers and fathers.

### Procedure

Participants were recruited through hard copy posters on campus, electronic advertisements (e.g., postings on Facebook groups, course websites, distribution to student club list serves, etc.), and classroom announcements. Interested participants contacted the lab via email to determine whether they were eligible for participation. Participants were eligible if they were currently enrolled in their 1st year of post-secondary school and lived in the surrounding area of the university. Participants were then assigned a unique identifier to complete the survey and sent a Qualtrics link to the consent form and baseline survey. Participants were surveyed during the first month of their first semester, and then again at 4 month and 8 month follow-ups (for a total of three assessments over 1 year). At each assessment, the survey took approximately 40 minutes to complete. Participants were provided with electronic gift cards (e.g., Amazon, Tim Hortons, Cineplex) for their time in the amounts of $10 at Time 1, $15 at Time 2, and $20 at Time 3.

The study was approved by the University Ethics Board, and all participants provided informed consent at each assessment point. Although research has consistently shown that asking students about self-injury does not increase risk of the behavior (Lewis et al., 2011; Muehlenkamp et al., 2010; Whitlock et al., 2013), several steps were taken to support the safety of participants. Participants were informed at recruitment and consent that they would be asked about NSSI engagement. Further, participants were informed that they could skip questions on the survey without penalty, and that they could withdraw from the study at any time. At any point during the assessment, participants also could click on a “Feeling Distressed” button, which directed them to a list of local mental health resources and supports. On the last survey page, participants were prompted to reflect and write about one good thing that happened to them on the previous day, which has been shown to induce positive mood in previous research (Seligman et al., 2005). At the end of each assessment, all participants received the list of local mental health resources, as well as a debriefing form with the principal investigator’s contact information.

### Missing Data

Missing data occurred in two primary circumstances: 1) missing data within the wave (i.e., participants did not answer all questions within the survey), and 2) missing data between waves (i.e., participants did not complete the survey for a particular wave). There was very little missing data within the wave (less than 1%). Overall, the study had very strong retention across the waves; 83% of participants completed all three waves of the survey, with 10% of participants completing two waves, and 7% only completing one wave. A MANOVA analysis demonstrated that participants who only completed one wave did not significantly differ from participants who completed two or three waves on any of the key study constructs, although these participants were more likely to be male. Given that missing data analysis indicated that study variables seemed to be missing at random, missing data was estimated using the full information maximum likelihood (FIML) estimation method. FIML was chosen due to its ability to retain cases with missing data, therefore avoiding potentially biased parameter estimates through pairwise and listwise deletion (Schafer & Graham, 2002).

### Measures

#### Demographic questionnaire

Participants completed a basic demographic questionnaire including questions about age, gender (one variable, with six response options of 0 = *male,* 1 = *female*, 2 = *transgender*, 3 = *unsure*, 4 = *prefer not to disclose,* 5 = *other*), parental education, and ethnicity. The present study limited analyses to males and females due to small sample sizes for the other gender identities, making gender a binary variable in the analyses (0 = *male*, 1 = *female*).

#### Depressive symptoms

Depressive symptoms were assessed using The Center for Epidemiological Studies Depression Scale–Revised (CESD-R, Eaton et al., 2004), which assesses depressive symptoms as outlined in the Diagnostic and Statistical Manual, fifth edition (DSM-5) (American Psychiatric Association, 2013). Participants were asked to rate 18 symptom statements (e.g., "nothing made me happy," "I was tired all the time") on a frequency scale from 0 = *not at all or less than one day* to 4 = *nearly everyday for 2 weeks*. The CESD-R has been validated in student samples, with good internal consistency, convergent validity, and divergent validity (Van Dam & Earleywine, 2011). The internal consistency of the CESD-R for the current study was excellent (α = .92, α = .94, and α = .95 for each of the three waves, respectively).

#### Perceived Stress Scale (PSS-10)

The PSS-10 was used to assess the degree to which students perceived their lives as currently stressful (Cohen & Williamson, 1988). The measure consists of 10 questions asking about participants’ feelings and thoughts over the past month (e.g., “how often have you felt that you were on top of things,” or “how often have you been upset because of something that happened unexpectedly?”). Responses range from 0 = *never* to 4 = *very often*. The PSS-10 has previously been found to be a reliable and valid self-report measure of perceived stress in university students, with strong internal consistency, convergent validity, and divergent validity (Roberti et al., 2006). The internal consistency of the PSS-10 was also strong in the present study (α = .85, α = .83, α = .83 for each of the three waves).

#### Self-Compassion Scale–Short Form (SCS-SF)

Self-compassion was assessed using the 12-item SCS-SF (Raes et al., 2011). Participants were asked to rate the frequency of 12 statements about themselves (e.g., "I try to see my failings as part of the human condition") from 1 = *almost never* to 5 = *almost always.* The original SCS includes six subscales: self-kindness, self-judgement, common humanity, isolation, mindfulness, and over-identification. Although the short form of the SCS has been found to have a strong correlation with the full scale (*r* ≥ 0.97), due to low reliability, the authors do not suggest using the SCS-SF’s subscales as identified in the full SCS (Raes et al., 2011). Therefore, the present study used the total self-compassion mean score from the SCS-SF. The SCS-SF has been found to have good internal consistency (*r* ≥ .86), test-retest reliability, and construct validity in community and clinical samples (Hayes et al., 2016; Raes, 2011; Raes et al., 2011). For the present study, internal consistency was good, with α = .80 for wave 1, α = .81 for wave 2, and α = .80 for wave 3.

#### Inventory of Statements About Self-Injury (ISAS)

NSSI engagement was assessed using the Inventory of Statements About Self-Injury (ISAS), developed by Klonsky and Glenn (2009). Participants were asked to estimate the number of times they engaged in a list of direct NSSI behaviors in the past 4 months (i.e., banging/hitting oneself, biting, burning, carving, cutting, rubbing skin against rough surfaces, and severe scratching). Although the ISAS typically assesses lifetime NSSI engagement, it has been adapted to include recent engagement as well (Heath et al., 2016; Victor & Klonsky, 2014). The number of times participants reported engaging in NSSI was summed on scale from 0 = *never* to 6 = *6 or more times*. Participants also indicated the extent to which 12 motivations for engaging in NSSI were relevant to them on a scale from 0 = *not at all relevant* to 2 = *very relevant*, including affect regulation, interpersonal boundaries, self-punishment, self-care, anti-dissociation, sensation-seeking, peer-bonding, interpersonal influence, toughness, marking distress, revenge seeking, and autonomy seeking. The ISAS has been shown to have good test-retest reliability and construct validity in university student populations (Klonsky & Glenn, 2009; Kortge et al., 2013).

### Plan of Analysis

Two models were built in Mplus 8 (Muthén & Muthén, 2017) to 1) examine associations among depressive symptoms, perceived stress, NSSI, and the possible mediating effects of self-compassion; and 2) to examine associations among depressive symptoms, perceived stress, and NSSI, moderated by self-compassion. The maximum likelihood estimation with robust standard errors (MLR) was selected due to its robustness to non-normality. Given this estimation, we used the MLR χ^2^ test statistic to compare fit across models (Muthén & Muthén, 2017). Overall model fit was evaluated using the comparative fit index (CFI), and the root mean square error of approximation (RMSEA; Bentler, 1995). Good model fit was assessed according to CFI values greater than .95 and RMSEA values less than .06, simultaneously (Hu & Bentler, 1999; Schreiber et al., 2006).

For each model, a model building sequence was completed as outlined in Mulder and Hamaker (2020), in which a RI-CLPM was fitted and then compared to a nested traditional cross-lagged panel model (CLPM). The RI-CLPM is an extension of the typical CLPM, which was introduced to account for stable, trait-like differences between individuals (Hamaker et al., 2015). The RI-CLPM does this by separating the within-person variance from the between-person variance by adding a random intercept for each construct in the model. Specifically, a latent random intercept was created for each variable, and then regressed onto the measure of that variable at each time point (e.g., a random intercept for depressive symptoms, regressed onto depressive symptoms measured at Time 1, Time 2, and Time 3). The residual variance provides an indication of the individuals’ deviation from their average levels of this construct, which is not accounted for in the CLPM model. Both the RI-CLPM and CLPM models tested also included associations among variables within each wave (i.e., concurrent associations), stability paths within each variable over time (i.e., autoregressive paths), and associations between variables over time (i.e., cross-lagged paths). We then tested whether constraining the model over time improved model fit, compared to when left unconstrained for time (i.e., a model in which autoregressive and cross-lagged paths were freely estimated).

For the moderation model, these above steps were completed again, but with self-compassion included as a moderator rather than a mediator. A grouping analysis was completed with self-compassion (high/low, split at the mean), to examine if there were group differences in the associations between depressive symptoms, perceived stress, and NSSI depending on students’ level of self-compassion. As also outlined in Mulder and Hamaker (2020), this was completed by comparing a multiple group version of the panel model in which there were no constraints across groups (e.g., students high in self-compassion and students low in self-compassion), to a model in which lagged regression coefficients were constrained to be identical across groups. Due to reports of gender differences in NSSI prevalence (Bresin & Schoenleber, 2015), gender was included as a time-invariant predictor in all models, with paths included from gender to observed variables within each assessment wave.^1^

## Results

### Preliminary

Descriptive analyses were run to explore the means of all study variables and the correlations among variables (Table 1). It was found that 25% of participants reported engaging in recent NSSI at Time 1, 23% of participants reported recent NSSI at Time 2, and 17.5% of participants reported recent NSSI at Time 3. Of participants who engaged in NSSI, the most common forms of NSSI were banging or hitting (51.3%), rubbing skin against rough surfaces (50%), and severe scratching (39.4%) at Time 1; banging or hitting (48.5%), biting (35.2%), and severe scratching (33.8%) at Time 2; and banging or hitting (54.3%), severe scratching (38.4%), and biting (36.1%) at Time 3. At each time point, the majority of participants who engaged in NSSI endorsed one or two methods (*M* = 1.53 at Time 1, *M* = 1.51 at Time 2, *M* = 1.63 at Time 3), and the average number of NSSI episodes in the past 4 months at each assessment was four. The most strongly endorsed motivations for engaging in NSSI were affect regulation, self-punishment, and anti-dissociation at Times 1 and 2, and affect regulation, self-punishment, and marking distress at Time 3.

**Table 1.** Correlations and Descriptive Statistics Among Study Variables.

Variables	1.	2.	3.	4.	5.	6.	7.	8.	9.	10.	11.	12.	13.
1. Gender	—	.087**	.113**	.119**	.257**	.247**	.259**	−.101**	−.114**	−.127**	.052	.023	.075*
2. Depressive Symptoms 1		—	.633**	.559**	.604**	.498**	.451**	−.464**	−.402**	−.401**	.279**	.249**	.206**
3. Depressive Symptoms 2			—	.613**	.469**	.566**	.467**	−.381**	−.451**	−.394**	.192**	.275**	.216**
4. Depression Symptoms 3				—	.438**	.457**	.566**	−.357**	−.393**	−.437**	.208**	.185**	.223**
5. Perceived Stress 1					—	.671**	.599**	−.543**	−.469**	−.449**	.224**	.182**	.188**
6. Perceived Stress 2						—	.659**	−.466**	−.537**	−.450**	.207**	.258**	.221**
7. Perceived Stress 3							—	−.438**	−.449**	−.562**	.185**	.221**	.226**
8. Self-Compassion 1								—	.699**	.647**	−.243**	−.207**	−.234**
9. Self-Compassion 2									—	.715**	−.244**	−.250**	−.237**
10. Self-Compassion 3										—	−.215**	−.208**	−.207**
11. NSSI Frequency 1											—	.552**	.504**
12. NSSI Frequency 2												—	.562**
13. NSSI Frequency 3													—
Mean	—	2.01	2.10	2.06	3.01	3.00	3.01	2.88	2.91	2.92	0.95	0.89	0.70
SD	—	0.74	0.82	0.86	0.66	0.63	0.66	0.67	0.67	0.66	1.95	1.91	1.74
Range	0–1	1–5	1–5	1–5	1–4.67	1–4.8	1–4.8	1.17–5	1.08–5	1.17–5	0–6	0–6	0–6

*Note*. Numbers correspond to time point. M = Standard Deviation. SD = standard deviation.

*p < .05. **p < .01.

#### Model 1. Longitudinal associations of depressive symptoms, perceived stress, and NSSI (mediated by self-compassion)

**Figure 1.**
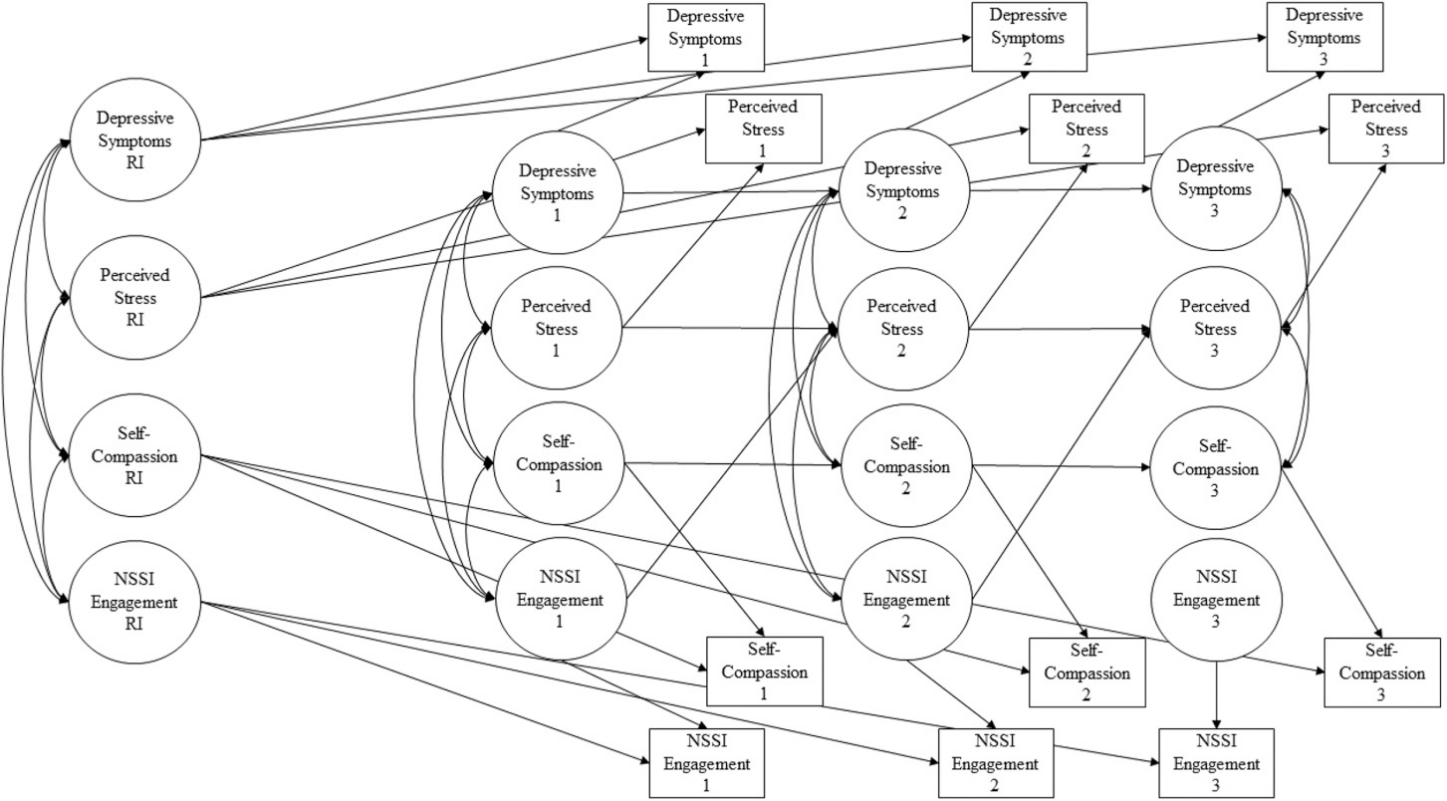
*RI-CLPM model. Note:* Numbers refer to time point. RI = random intercept. Only significant paths are displayed. Gender is not included in the figure for simplicity.

Model fit indices for all models tested are provided in Table 2. We first ran a RI-CLPM model of depressive symptoms, perceived stress, self-compassion, and NSSI, with gender predicting observed variables across all time points (see Figure 1) for RI-CPLM model. This model was compared to a nested traditional CLPM, in which the variances of the random intercept and their covariances were constrained to zero. The χ^2^ Difference Test of Relative Fit indicated that the RI-CLPM model improved fit compared to the traditional CLPM, suggesting that there were stable, between-person differences that should be accounted for, Δχ^2^ = 160.28(10), *p* < .001. In line with best-practice recommendations (Hamaker et al., 2015), we then compared whether the RI-CLPM model constrained for time improved model fit relative to the model unconstrained for time, given that time invariant models can improve interpretability, reliability, and statistical power (Hamaker et al., 2015). The χ^2^ Difference Test of Relative Fit indicated that the model constrained for time did not worsen model fit compared to the model unconstrained for time, RI-CLPM, Δχ^2^ = 8.73(16), *p* = .92. Given a lack of significance, this implies that constraints over time were tenable due to time-invariant processes across the data collection phases (Mulder & Hamaker, 2020). Additionally, an examination of additional fit indices demonstrated that the model constrained for time had a lower AIC (27500.95) and BIC (27897.96), compared to the unconstrained model (27522.65 AIC, 28000.07 BIC) (Mulder & Hamaker, 2020). Therefore, the remainder of the interpretations are based off the RI-CLPM model, constrained over time, CFI = 1.00, RMSEA = .00, 95% CI [.000, .014]. The full standardized parameter estimates from this model can be found in Table 3.

**Table 2.** Model Fit Indices for Cross-Lagged Panel Models and Random Intercept Cross-Lagged Panel Models.

Model	χ^2^(df)	RMSEA [90% CI]	CFI	TLI	SRMR	AIC	BIC	Model Comparison: Δχ^2^*(*Δ*df)*
Depressive symptoms, perceived stress, and NSSI, mediated by self-compassion								
CLPM	197.918 (19)	.091 [.080, .103]	.967	.865	.032	27729.889	28157.060	
RI-CLPM	10.204 (9)	.011 [.000, .036]	1.000	.998	.008	27522.647	28000.073	Δχ^2^ = 160.28(10), *p* < .001
** RI-CLPM, time constrained**	**18.288 (25)**	**.000 [.000, .014]**	**1.000**	**1.000**	**.013**	**27500.947**	**27897.964**	**Δχ^2^ = 8.73(16),** ***p* = .92**
Depressive symptoms, perceived stress, and NSSI, moderated by self-compassion								
CLPM	146.254 (12)	.100 [.086, .115]	.959	.847	.037	23597.803	23864.156	
RI-CLPM	10.074 (6)	.025 [.000, .050]	.999	.991	.011	23430.830	23727.336	Δχ2 = 109.47(6), *p* < .001
** RI-CLPM, time constrained**	**11.035 (15)**	**.000 [.000, .020]**	**1.000**	**1.000**	**.013**	**23416.168**	**23667.445**	**Δχ2 = 2.55 (9),** ***p* = .98**
RI-CLPM, time constrained, groups unconstrained	37.476 (39)	.000 [.000, .028]	1.000	1.000	.025	23040.852	23498.176	
RI-CLPM, time unconstrained, groups constrained	40.585 (48)	.000 [.000, .020]	1.000	1.000	.028	23028.297	23440.391	Δχ2 = 3.98(9), *p* = .91

*Note.* CLPM = cross-lagged panel model, RI-CLPM = random intercept cross-lagged panel model. Model comparison represents difference in model fit between the model and the model above. Bolded models are best fitting.

**Table 3.** Parameter Estimates for Final RI-CLPM.

Parameter	B	SE	p	95% CI
Covariances				
Between-Person Associations				
Depression—Perceived Stress	.763	.031	.000**	[.702, .823]
Depression—NSSI	.396	.055	.000**	[.288, .504]
Depression—Self-Compassion	−.603	.039	.000**	[−.680, −.527]
Perceived Stress—Self-Compassion	−.710	.036	.000**	[−.780, −.640]
Perceived Stress—NSSI	.331	.049	.000**	[.234, .427]
NSSI—Self-Compassion	−.368	.048	.000**	[−.462, −.275]
Within-Person Associations				
Depression 1—Perceived Stress 1	.389	.053	.000**	[.286, .493]
Depression 1—NSSI 1	.184	.058	.001**	[.071, .297]
Depression 1—Self-Compassion 1	−.255	.054	.000**	[−.360, −.149]
Perceived Stress 1—Self-Compassion 1	−.303	.057	.000**	[−.415, −.192]
Perceived Stress 1—NSSI 1	.144	.052	.005**	[.043, .245]
NSSI 1—Self-Compassion 1	−.110	.052	.034*	[−.212, −.008]
Depression 2—Perceived Stress2	.327	.052	.000**	[.226, .428]
Depression 2—NSSI 2	.137	.060	.022*	[.020, .255]
Depression 2—Self-Compassion 2	−.253	.051	.000**	[−.353, −.152]
Perceived Stress 2—Self-Compassion 2	−.260	.063	.000**	[−.383, −.137]
Perceived Stress 2—NSSI 2	.203	.054	.000**	[.096, .309]
NSSI 2—Self-Compassion 2	−.115	.060	.056	[−.233, .003]
Depression 3—Perceived Stress 3	.346	.036	.000**	[.276, .417]
Depression 3—NSSI 3	.068	.046	.139	[−.022, .157]
Depression 3—Self-Compassion 3	−.190	.043	.000**	[−.274, −.106]
Perceived Stress 3—Self-Compassion 3	−.342	.043	.000**	[−.427, −.257]
Perceived Stress 3—NSSI 3	.075	.052	.151	[−.027, .177]
NSSI 3—Self-Compassion 3	.008	.050	.869	[−.089, .106]
Gender and Observed Variables				
Gender → Depression 1	.111	.033	.001**	[.046, .175]
Gender → Depression 2	.099	.030	.001**	[.041, .158]
Gender → Depression 3	.096	.029	.001**	[.040, .153]
Gender → Perceived Stress 1	.232	.029	.000**	[.176, .288]
Gender → Perceived Stress 2	.236	.028	.000**	[.181, .291]
Gender → Perceived Stress 3	.231	.028	.000**	[.177, .285]
Gender → Self-Compassion 1	−.093	.029	.001**	[−.150, −.037]
Gender → Self-Compassion 2	−.095	.029	.001**	[−.151, −.038]
Gender → Self-Compassion 3	−.096	.029	.001**	[−.153, −.038]
Gender → NSSI 1	.041	.026	.114	[−.010, .091]
Gender → NSSI 2	.041	.026	.114	[−.010, .093]
Gender → NSSI 3	.044	.028	.113	[−.011, .099]
Within-Person Stability paths				
Depression 1 → Depression 2	.206	.056	.000**	[.096, .317]
Depression 2 → Depression 3	.243	.067	.000**	[.111, .374]
Perceived Stress 1 → Perceived Stress 2	.176	.066	.008**	[.046, .305]
Perceived Stress 2 → Perceived Stress 3	.162	.065	.013*	[.034, .289]
Self-Compassion 1 → Self-Compassion 2	.226	.067	.001**	[.094, .358]
Self-Compassion 2 → Self-Compassion 3	.226	.077	.004**	[.074, .377]
NSSI 1 → NSSI 2	.102	.084	.222	[−.062, .266]
NSSI 2 → NSSI 3	.115	.097	.237	[−.076, .305]
Within-Person T1, T2 cross-lags				
Perceived Stress 1 → Depression 2	.052	.045	.248	[−.036, .140]
Self-Compassion 1 → Depression 2	−.056	.045	.220	[−.145, .033]
NSSI 1 → Depression 2	−.043	.041	.301	[−.123, .038]
Depression 1 → Perceived Stress 2	.057	.044	.196	[−.029, .143]
Self-Compassion 1 → Perceived Stress 2	−.055	.066	.405	[−.183, .074]
NSSI 1 → Perceived Stress 2	.091	.042	.029*	[.009, .174]
Depression 1 → Self-Compassion 2	−.022	.041	.588	[−.104, .059]
Perceived Stress 1 → Self-Compassion 2	−.009	.053	.859	[−.113, .094]
NSSI 1→ Self-Compassion 2	−.066	.044	.136	[−.152, .021]
Depression 1 → NSSI 2	.046	.041	.262	[−.035, .127]
Perceived Stress 1 → NSSI 2	.035	.047	.452	[−.057, .128]
Self-Compassion 1 → NSSI 2	−.012	.046	. 791	[−.103, .079]
Within-Person T2, T3 cross-lags				
Perceived Stress 2 → Depression 3	.047	.041	.254	[−.034, .129]
Self-Compassion 2 → Depression 3	−.051	.042	.229	[−.134, .032]
NSSI 2 → Depression 3	−.039	.037	.300	[−.112, .034]
Depression 2 → Perceived Stress 3	.068	.053	.199	[−.036, .171]
Self-Compassion 2 → Perceived Stress 3	−.051	.061	.410	[−.171, .070]
NSSI 2 → Perceived Stress 3	.084	.039	.031*	[.008, .161]
Depression 2 → Self-Compassion 3	−.029	.053	.588	[−.133, .075]
Perceived Stress 2 → Self-Compassion 3	−.009	.052	.859	[−.112, .093]
NSSI 2 → Self-Compassion 3	−.065	.043	.132	[−.150, .020]
Depression 2 → NSSI 3	.067	.060	.262	[−.050, .184]
Perceived Stress 2 → NSSI 3	.040	.053	.453	[−.064, .144]
Self-Compassion 2 → NSSI 3	−.014	.052	.791	[−.117, .089]

*Note*. Numbers after variables indicate assessment wave; *B* = standardized coefficient; *SE* = standard error. CI = confidence intervals. Depression refers to depressive symptoms.

##### Between-person level

The covariances among random intercepts for depressive symptoms, perceived stress, self-compassion, and NSSI were all significant at the *p* < .001 level. Higher levels of trait-like depressive symptoms were associated with higher levels of trait-like perceived stress, lower levels of trait-like self-compassion, and higher levels of trait-like NSSI engagement. Higher levels of trait-like perceived stress were associated with lower levels of trait-like self-compassion, and higher levels of trait-like NSSI engagement. Lastly lower levels of trait-like self-compassion were associated with higher levels of trait-like NSSI engagement.

##### Within-person level

At Time 1, all concurrent associations were significant at the within-person level. The within-time covariances among the construct residuals (see Table 3) demonstrated that at times when individuals experienced higher than typical levels of depressive symptoms, they also experienced higher than typical levels of perceived stress at Times 2 and 3, lower than typical levels of self-compassion at Times 2 and 3, and higher than typical levels of NSSI engagement at Time 2. At times when students had higher than typical levels of perceived stress, they also had lower than typical levels of self-compassion at Times 2 and 3, and higher levels of NSSI at Time 2.

##### Cross-Lagged Paths

NSSI at Time 1 predicted increased perceived stress at Time 2 (β = .091, *SE* = .042, *p* = .029, 95% CI [.009, .174), and NSSI at Time 2 predicted increased perceived stress at Time 3 (β = .084, *SE* = .039, *p* = .031, 95% CI [.008, .161]). As interpreted by (Hamaker et al., 2015), when an individual reported higher than their typical level of NSSI engagement, they also reported intraindividual increases in perceived stress, at the subsequent assessment point. This effect was quite small from Time 1 to Time 2, and Time 2 to Time 3; for example, for every additional NSSI incident at Time 1, students were likely to report a .091 standard deviation increase in perceived stress at Time 2 (a similarly sized effect was found from NSSI at Time 2 to perceived stress at Time 3). No other cross-lagged paths were statistically significant.

### Model 2: Longitudinal associations of depressive symptoms, perceived stress, and NSSI (moderated by self-compassion)

We also computed a RI-CLPM of depressive symptoms, perceived stress, and NSSI, with gender predicting observed variables, and with self-compassion as a moderator (rather than a mediator). Model fit indices can be found in Table 2. This model was compared to a traditional CLPM, in which the variances of the random intercept and their covariances were constrained to zero. The χ^2^ Difference Test of Relative Fit indicated that the RI-CLPM model improved fit compared to the traditional CLPM, indicating that there were stable, between-person differences in the variables, Δχ^2^ = 109.47(6), *p* < .001. We then tested if we could constrain the RI-CLPM paths over time; the χ^2^ Difference Test of Relative Fit indicated that the RI-CLPM model constrained over time did not worsen model fit compared to the unconstrained over time RI-CLPM, Δχ^2^ = 2.55(9), *p* = .98, implying that constraints over time were tenable due to time-invariant processes (Mulder & Hamaker, 2020). Additionally, the model constrained by time had a lower AIC (23416.17) and BIC (23667.45), compared to the unconstrained model (23430.83 AIC, 23727.34 BIC) (Mulder & Hamaker, 2020). Building upon the time constrained RI-CLPM model, there was no significant difference in model fit in the grouping analysis, in which we compared an unconstrained lagged parameters model to a model in which lagged parameters were constrained to be equal among the two self-compassion groups, Δχ^2^ = 3.98(9), *p* = .91. As the constraint could be imposed across groups, this implies that the lagged coefficients did not significantly differ between students with low self-compassion and students with high self-compassion.

## Discussion

Many emerging adults report experiencing depressive symptoms and stress during the challenging post-secondary years (American College Health Association, 2019), and rates of NSSI are also elevated during this time (Swannell et al., 2014, Wester et al., 2018). Although depressive symptoms and stress have been implicated as potential risk factors for NSSI (Garisch & Wilson, 2015; Liu et al., 2016; Wilcox et al., 2012), there is limited multi-wave longitudinal work assessing the nature of these associations, or potential mediating and mitigating factors. Building on recent theoretical work, which suggests that positive self-beliefs may serve as important barriers to NSSI engagement (Hooley & Franklin, 2017), we sought to clarify the longitudinal associations among depressive symptoms, perceived stress, and NSSI, taking into account the potential role of self-compassion. We expected that there would be stable trait-like between-person differences in study variables, and that individuals who were higher on trait depressive symptoms and stress, and low on self-compassion, would also have higher trait-like engagement in NSSI. We also predicted that when participants reported higher depressive symptoms and perceived stress, they would also report lower levels of self-compassion and higher levels of NSSI over time (relative to that student’s typical levels on these variables). Uniquely taking into account these between-person effects, we also examined the within-person lagged effects over time. Specifically, we examined whether intraindividual increases in depressive symptoms and perceived stress predicted intraindividual increases in NSSI over time (or the reverse), and whether these associations were mediated and/or moderated by self-compassion. As expected, all of the variables had trait-like attributes which were associated, and at times when individuals were at higher risk on one of these variables, they tended to be higher in risk on the other variables as well. An examination of within-person effects revealed that only NSSI predicted increasing stress over time, but this effect was small. Our findings underscore why disentangling between and within-person variance is critical to elucidate the nature of associations among depressive symptoms, perceived stress, self-compassion, and NSSI.

Consistent with study hypotheses, depressive symptoms, perceived stress, self-compassion, and NSSI all had stable, trait-like attributes at the between-person level, suggesting individuals tended to score consistently higher or lower on these measures, relative to others. These trait-like attributes also were associated, such that individuals who tended to score higher on depressive symptoms, perceived stress, and lower on self-compassion (on average), also tended to have higher trait-engagement in NSSI. Further, at times when individuals rated themselves as higher risk on one of these variables (e.g., higher depressive symptoms, perceived stress, NSSI, and lower self-compassion than their typical levels), they tended to be higher in risk on the other variables as well. These findings are consistent with previous studies, which have identified trait-like stability for depressive symptoms (Masselink et al., 2018), perceived stress (Miller et al., 2020; van Eck et al., 1998), self-compassion (Neff, 2003), and NSSI (Barrocas et al., 2014). Further, findings are consistent with research demonstrating cross-sectional associations among depressive symptoms, perceived stress, self-compassion, and NSSI in the literature (Hankin & Abela, 2011; Kiekens et al., 2015; Kokaliari et al., 2017; Lathren et al., 2019).

After accounting for stable between-person differences, we sought to understand the relations between depressive symptoms, perceived stress, self-compassion, and NSSI at the within-person level. Inconsistent with study hypotheses, higher depressive symptoms and perceived stress did not predict increases in NSSI within individuals over time, nor did self-compassion (i.e., no indirect effects). Thus, our study uniquely demonstrates that although depressive symptoms, perceived stress, and lower self-compassion may co-occur with NSSI, they may not temporally predict increased NSSI engagement over time. There may be other unmeasured factors that contribute to higher levels of depressive symptoms, perceived stress, and NSSI, and lower levels of self-compassion. For example, emotion dysregulation (i.e., difficulty modulating one’s emotional responses) (Gross, 2015) has been widely implicated in the development of depressive symptoms (Joormann & Stanton, 2016; Schäfer et al., 2017), perceived stress (Wang & Saudino, 2011), and NSSI engagement (You et al., 2018). It is possible then that another factor, such as emotion dysregulation, may be similarly driving an individual’s risk for depressive symptoms, perceived stress, lower self-compassion, and NSSI.

Our finding that increases in NSSI predicted higher levels of perceived stress over time, after taking into account between-person effects, is especially novel given that our study is the first to disentangle between and within-person variance to examine associations among depressive symptoms, perceived stress, and NSSI over time. Although this effect was quite small, there are a number of potential explanations for why NSSI may lead to heightened stress. Engaging in NSSI may undermine the development of emotional regulation or alternative coping strategies, leading to higher levels of stress over time (Ewing et al., 2019; Hasking et al., 2017; Zelkowitz et al., 2016). Consistent with the stress generation hypothesis (Burke et al., 2015; March-Llanes et al., 2017), NSSI could also lead to heightened exposure to stressors over time (e.g., engaging in NSSI may lead to conflict with family, friends, and romantic partners who disapprove of the behavior). Previous research has suggested that NSSI influences subsequent stress in romantic and parent-child relationships, and the occurrence of other interpersonal stressful events (Burke et al., 2015; Miller et al., 2018). Additionally, engaging in NSSI in and of itself may also be stressful, connected to feelings of shame (Sheehy et al., 2019) and self-stigma (Piccirillo et al., 2020). These possibilities should be explored in future research, in studies on the association between perceived stress and NSSI.

Due to reports of potential gender differences in NSSI prevalence (Bresin & Schoenleber, 2015), we examined gender as a potential covariate influencing the within-person associations between depressive symptoms, perceived stress, self-compassion, and NSSI. Gender significantly predicted depressive symptoms, perceived stress, and self-compassion at all time points, such that being female predicted higher levels of depressive symptoms, higher levels of perceived stress, and lower levels of self-compassion at each time point. In contrast, gender was not significantly associated with NSSI at any time point. Previous studies have also found similar patterns with gender differences in depressive symptoms (Frye & Liem, 2011), perceived stress (Anbumalar et al., 2017), and self-compassion (Yarnell et al., 2015), and underscore that females may be particularly vulnerable for these mental health concerns in the post-secondary context.

### Limitations and Directions for Future Research

Despite the many strengths of the present study, including the use of a multi-wave longitudinal approach and a large diverse sample, there are some important limitations worth nothing. First, the present sample consisted of largely female, upper-class East Asian, South Asian, and Caucasian students. Although our sample was representative of the student population from which it was drawn (and 80% were not Caucasian), findings may not generalize to adolescent, adult, clinical, and more ethnically diverse samples. Additionally, we did not specifically examine ethnicity as a moderator of the proposed associations; however, this represents an important extension for future research to facilitate a better understanding of the experiences of racialized students, while taking into account the stressors specifically relevant for these students (e.g., discrimination and victimization).

Another limitation of the present study is the possibility of self-selection bias. Participants were recruited broadly across campus to participate in a study on student experiences in the 1st year of university. We did not specifically recruit for a study on NSSI, but students who were willing to participate in a study on student experiences may differ from students who did not choose to participate. Moreover, all assessments in the present study utilized retrospective self-report, which is subject to errors in recall. To mitigate this issue, we adopted shorter assessment intervals for recalling behaviors (e.g., 4 months of NSSI engagement), rather than lifetime NSSI assessment (which is often the standard in the field, see Fox et al., 2015 for a review). Ecological momentary assessment based approaches (i.e., EMA, assessing participants multiple times a day over several days) have also been shown to increase accuracy of self-reports, and represent an important extension for future research (Russell & Gajos, 2020). Future research could combine EMA and longitudinal assessment together (a technique known as measurement burst sampling; Cho et al., 2019) to examine how these daily processes contribute to more enduring developmental change and stability in these constructs over time. However, it is important to note that a limitation of all self-report data is shared method variance (meaning that all measures were completed by the same individual, so other individual unmeasured factors could have contributed to participants’ scores on all the study measures; e.g., mood). In the future, researchers could incorporate additional measures to corroborate self-report findings (e.g., collecting exam schedules to test for academic stressors), as well as utilize ambulatory data (e.g., sensory data like heart rate, respiration to assess stressors and/or emotions, rather than perceived stress and emotions) (Trull & Ebner-Priemer, 2013).

It is important to highlight that our prevalence rates of recent NSSI were in the upper-range of estimates, but not inconsistent with other studies which have used similar assessment approaches (Greene et al., 2019; Kiekens et al., 2019). A meta-analysis on the factors associated with NSSI reporting demonstrated that several factors may increase NSSI disclosure rates (Swannell et al., 2014), such as using checklist measures like the ISAS, measures that are self-administered, and measures that offer a high degree of confidentiality (like in the present study). Adding EMA assessments of this behavior may offer additional advantages in accuracy of NSSI reporting.

Our study also focused on predictors of frequency of NSSI engagement, regardless of underlying motivations for NSSI. However, there is likely quite a bit of heterogeneity among individuals who engage in NSSI. For example, the role of self-compassion in NSSI engagement may vary depending on the motivations for NSSI. Self-compassion may be more relevant to understanding NSSI behaviors driven primarily by the need to self-punish, rather than engaging in NSSI for other motivations (such as emotion regulation). Future research may benefit from utilizing more person-centered approaches that take into account heterogeneity in NSSI engagement.

Finally, our study is correlational in nature, and therefore causality cannot be inferred. However, our study does explore directionality of effects, and adds to the literature on the associations among depressive symptoms, stress, self-compassion, and NSSI over time. It also is important to note that the significant cross-lagged effect observed in the path model from NSSI to perceived stress was quite small, and so the finding should be interpreted cautiously. Our model controlled for stable between-person differences, stability paths, and concurrent associations among variables at each time point, to examine within-person cross-lagged effects over time, so to some extent it would be expected that these effects would be small. For example, Adachi and Willoughby (2015) suggest that in the context of CLPM, a score of 0.05 would be a more appropriate measure of a small effect, relative to standard cut-offs (e.g., .10). Additionally, our findings are not inconsistent with other literature on longitudinal predictors of NSSI which have often reported small effect sizes (Fox et al., 2015), particularly studies using CLPM modeling (Baetens et al., 2015; Wu et al., 2019; You et al., 2012). Nevertheless, it is unclear to what extent the finding that NSSI may lead to increased perceived stress has practical and clinical utility, given the small effect size. Additional research is necessary to further explore the effects of NSSI on stress over time.

### Conclusions and Implications

The emerging adult years are a period of increased vulnerability for mental health challenges, including depressive symptoms, stress, and NSSI. Recently, it has been suggested that increased levels of depressive symptoms and perceived stress during the post-secondary years may lead to increased risk for NSSI, but longitudinal research on the nature of associations among depressive symptoms, perceived stress, and NSSI are lacking. Cross-sectional findings also have implicated self-compassion as a potential mediator of the associations between depressive symptoms, perceived stress, and NSSI and as a potential protective factor preventing students from engaging in NSSI; however, the potential mediating and/or moderating effects of self-compassion have yet to be tested longitudinally. Using a RI-CLPM approach, we found that depressive symptoms, perceived stress, self-compassion, and NSSI had stable, trait-like differences, which were associated. Individuals who tended to report higher trait-levels of depressive symptoms and perceived stress, and lower levels of self-compassion—on average—also reported trait-like engagement in NSSI, relative to others. We also found that at times when individuals were higher on one of these variables, they tended to be higher on these other variables. These findings suggest that individuals who engage in NSSI may be vulnerable for co-morbid, elevated levels of depressive symptoms and perceived stress, and low self-compassion. These results underscore the necessity of a comprehensive assessment approach that takes into account the totality of an individual’s level of functioning so that an appropriate care plan can be developed.

Although we expected depressive symptoms and perceived stress to predict increased risk for NSSI over time indirectly through self-compassion, this hypothesis was not supported. Self-compassion also did not significantly moderate the connections among depressive symptoms, perceived stress, and NSSI. Instead, taking into account between-person effects, it was found that NSSI predicted increased perceived stress over time, and not the reverse. However, it is important to note that this effect was quite small. Nevertheless, these findings lend some support for the stress-generation hypothesis (Burke et al., 2015; March-Llanes et al., 2017), which suggest that engaging in NSSI may lead to heightened stress over time among students. Thus, identifying sources of stress stemming from NSSI engagement (e.g., increased interpersonal conflict, shame and guilt, etc.) may represent an important future research direction. Mitigating these sources of stress may be helpful as part of NSSI support and intervention for students.

## Note

1 We also ran the models with gender predicting random intercepts instead of observed variables, and a model without gender included as a covariate; the pattern of results did not vary.
